# Supplementary material for: Development and validation of the arabic version of the social-ecological model questionnaire for patients undergoing maintenance hemodialysis
Source: PLoS One. 2025 Oct 16;20(10):e0333740. doi: 10.1371/journal.pone.0333740 (PMC12530521; doi:10.1371/journal.pone.0333740)
Supplement: S1 File — (DOCX) [file pone.0333740.s001.docx]

**S1. English Version of Social-Ecological Model Questionnaire for Hemodialysis Patients**

This questionnaire was developed to assess the multi-level factors influencing the experiences and outcomes of patients undergoing maintenance hemodialysis within the framework of the Social-Ecological Model. The Social-Ecological Model emphasizes the interplay between individual, interpersonal, organizational, community, and policy levels in shaping health behaviors and outcomes. Recognizing the significant external challenges faced by maintenance hemodialysis patients, this tool focuses on four key domains: interpersonal, organizational, community, and policy. Each domain includes 10 items, ensuring a balanced representation of external influences while maintaining practicality for patients who may experience fatigue or time constraints. The questionnaire was designed to align with the cultural and contextual realities of Arabic-speaking populations, where family, community, and healthcare systems play a critical role in patient care. By excluding the intrapersonal domain, socio-demographic variables were used as proxies for individual-level factors. This tool aims to provide a comprehensive understanding of the systemic and environmental determinants affecting maintenance hemodialysis patients, with potential applications in research, clinical practice, and policy development.

**Part 1: Support at the family and interpersonal levels for hemodialysis patients**

The support of loved ones plays an essential role in coping with hemodialysis. We aim to assess the extent of emotional and practical support you receive from your family members during hemodialysis journey.

Rate each statement based on your experience.

| **Statements** | **Strongly disagree**  **(1)** | **Disagree**  **(2)** | **Neutral**  **(3)** | **Agree**  **(4)** | **Strongly agree**  **(5)** |
| --- | --- | --- | --- | --- | --- |
| 1. My family members support me emotionally during my journey with hemodialysis. |  |  |  |  |  |
| 1. My family members help me with my daily needs and treatment. |  |  |  |  |  |
| 1. I can share my fears about hemodialysis with my family members. |  |  |  |  |  |
| 1. My family members are well-informed about my conditions. |  |  |  |  |  |
| 1. I can openly talk to my family members about my challenges with hemodialysis. |  |  |  |  |  |
| 1. My family members encourage me to do healthy activities. |  |  |  |  |  |
| 1. My family members support my treatment decisions. |  |  |  |  |  |
| 1. My family understands my limits related to hemodialysis. |  |  |  |  |  |
| 1. My family members adjust their lives to help me. |  |  |  |  |  |
| 1. My family members actively support my well-being. |  |  |  |  |  |

**Part 2: Community-level support for hemodialysis patients.**

Navigating hemodialysis is not just an individual journey but also one that is significantly influenced by the understanding and support of the community. Your insights into the role of the community in facilitating care, awareness, and empathy for hemodialysis patients are invaluable.

Indicate your level of agreement with the following statements:

| **Statements** | **Strongly disagree (1)** | **Disagree (2)** | **Neutral (3)** | **Agree (4)** | **Strongly agree**  **(5)** |
| --- | --- | --- | --- | --- | --- |
| 1. I feel that my community provides me with the emotional support I need to cope with hemodialysis. |  |  |  |  |  |
| 1. I believe that community awareness about hemodialysis has improved my quality of life. |  |  |  |  |  |
| 1. I receive practical help from community members, which makes managing my hemodialysis treatment easier. |  |  |  |  |  |
| 1. I feel less isolated because of the social connections made within my community. |  |  |  |  |  |
| 1. My community offers educational resources that help me understand my condition and treatment better. |  |  |  |  |  |
| 1. I find that peer support from other hemodialysis patients in my community is beneficial to my mental health. |  |  |  |  |  |
| 1. I feel that my community is empathetic toward the challenges I face as a hemodialysis patient. |  |  |  |  |  |
| 1. I believe that community initiatives have made it easier for me to access hemodialysis care. |  |  |  |  |  |
| 1. I feel encouraged by the community's involvement in raising awareness about kidney disease and hemodialysis. |  |  |  |  |  |
| 1. I find that community support has positively influenced my adherence to my hemodialysis treatment regimen. |  |  |  |  |  |

**Part 3: Organizational/institutional level support for Hemodialysis patients**

Institutes such as hospitals and organizational involvement can profoundly influence the experience of individuals navigating hemodialysis. We want to understand your perception of the support extended by the hospital or dialysis center and the social or community organizations at large regarding hemodialysis care. Express your level of agreement with the following statements:

| **Statements** | **Strongly disagree (1)** | **Disagree (2)** | **Neutral (3)** | **Agree (4)** | **Strongly agree (5)** |
| --- | --- | --- | --- | --- | --- |
| 1. The hospital/dialysis center provides me with comprehensive education and resources to better understand my condition and treatment. |  |  |  |  |  |
| 1. I feel that the staff at the hospital/dialysis center is empathetic and responsive to my needs as a hemodialysis patient. |  |  |  |  |  |
| 1. The hospital/dialysis center has support groups or programs that help me cope with the emotional challenges of undergoing hemodialysis. |  |  |  |  |  |
| 1. I believe that the hospital/dialysis center collaborates effectively with community organizations to raise awareness about kidney disease and hemodialysis. |  |  |  |  |  |
| 1. The hospital/dialysis center has made efforts to involve my family and loved ones in my care and treatment plan. |  |  |  |  |  |
| 1. I am satisfied with the level of care and attention I receive from the hospital/dialysis center during my hemodialysis sessions. |  |  |  |  |  |
| 1. I feel that the community organizations in my area are actively working to promote better understanding and support for hemodialysis patients. |  |  |  |  |  |
| 1. I believe that the hospital/dialysis center and community organizations are committed to improving the overall quality of life for hemodialysis patients. |  |  |  |  |  |
| 1. I feel that the hospital/dialysis center and community organizations have created a supportive environment that fosters hope and positivity for hemodialysis patients. |  |  |  |  |  |
| 1. The hospital/dialysis center has provided me with access to counselling or mental health services to help me cope with the psychological impact of hemodialysis. |  |  |  |  |  |

**Part 4: Supportive policy for hemodialysis patients**

Policies play an essential role in shaping the care and support that hemodialysis patients receive. We need to assess how you perceive the efficacy of policies in addressing hemodialysis concerns.

Express your level of agreement with the following statements:

| **Statements** | **Strongly disagree (1)** | **Disagree (2)** | **Neutral (3)** | **Agree (4)** | **Strongly agree (5)** |
| --- | --- | --- | --- | --- | --- |
| 1. I believe that current policies ensure that I receive timely and adequate hemodialysis treatment. |  |  |  |  |  |
| 1. I feel that the policies in place provide sufficient access to necessary medical supplies for my hemodialysis sessions. |  |  |  |  |  |
| 1. I am confident that the policies support the availability of trained medical personnel to assist with my hemodialysis. |  |  |  |  |  |
| 1. I think that the policies effectively address the need for psychological support for hemodialysis patients. |  |  |  |  |  |
| 1. I feel that the policies ensure that I have access to nutritional counselling and support. |  |  |  |  |  |
| 1. I believe that the policies promote awareness and understanding of hemodialysis within the community. |  |  |  |  |  |
| 1. I feel that the policies in place protect my rights as a hemodialysis patient. |  |  |  |  |  |
| 1. I think that the policies facilitate the coordination between hospitals and community organizations to support hemodialysis patients. |  |  |  |  |  |
| 1. I feel that the policies ensure that I receive equitable treatment regardless of my socio-economic status. |  |  |  |  |  |
| 1. I believe that the policies are responsive to the changing needs and challenges faced by hemodialysis patients. |  |  |  |  |  |

**Thank You for Your Participation**
