# Supplementary material for: Development and validation of the arabic version of the social-ecological model questionnaire for patients undergoing maintenance hemodialysis
Source: PLoS One. 2025 Oct 16;20(10):e0333740. doi: 10.1371/journal.pone.0333740 (PMC12530521; doi:10.1371/journal.pone.0333740)
Supplement: S2 File — (DOCX) [file pone.0333740.s002.docx]

**S2. Arabic version of the Socio-Ecological Model Questionnaire for Hemodialysis Patients**

**النسخة العربية المطورة من استبيان النموذج الاجتماعي البيئي لمرضى الغسيل الكلوي**

تم تطوير هذا الاستبيان لتقييم العوامل متعددة المستويات التي تؤثر على تجارب ونتائج المرضى الذين يخضعون للغسيل الكلوي، وذلك ضمن إطار النموذج الاجتماعي البيئي. يؤكد هذا النموذج على التفاعل بين المستويات الفردية، والبين شخصية، والتنظيمية، والمجتمعية، والسياساتية في تشكيل السلوكيات الصحية والنتائج المرتبطة بها. ونظرًا للتحديات الخارجية الكبيرة التي يواجهها مرضى الغسيل الكلوي، تركز هذا الأداة على أربعة محاور رئيسية: البين شخصي، والتنظيمي، والمجتمعي، والسياساتي. يتضمن كل محور عشرة بنود، مما يضمن تمثيلًا متوازنًا للعوامل الخارجية مع مراعاة الجوانب العملية للمرضى الذين قد يعانون من الإرهاق أو ضيق الوقت.

تم تصميم الاستبيان ليتماشى مع السياقات الثقافية والواقعية للمجتمعات الناطقة باللغة العربية، حيث تلعب الأسرة والمجتمع والنظم الصحية دورًا محوريًا في رعاية المرضى. وبما أن البعد الفردي لم يُدرج في المحاور الأساسية، فقد تم استخدام المتغيرات الاجتماعية والديموغرافية كبدائل للعوامل الفردية. تهدف هذه الأداة إلى توفير فهم شامل للعوامل النظامية والبيئية المؤثرة على مرضى الغسيل الكلوي، مع إمكانيات تطبيقها في البحث العلمي والممارسات السريرية وتطوير السياسات الصحية.

**الجزء الأول: الدعم على مستوى الأسرة والعلاقات الشخصية لمرضى الغسيل الكلوي**

يلعب دعم الأحباء دورًا أساسيًا في التكيف مع الغسيل الكلوي. لذاك نهدف إلى قياس مدى الدعم العاطفي والعملي الذي تتلقاه من أفراد عائلتك خلال هذه الرحلة.

قيّم كل عبارة بناءً على تجربتك.

| **العبارة** | | **غير موافق بشدة**  **(1)** | **غير موافق**  **(2)** | **محايد**  **(3)** | **موافق**  **(4)** | **موافق بشدة**  **(5)** |
| --- | --- | --- | --- | --- | --- | --- |
| 1. | يدعمني أفراد عائلتي عاطفيًا خلال رحلتي مع الغسيل الكلوي. |  |  |  |  |  |
| 2. | يساعدني أفراد عائلتي في علاجي تلبية احتياجاتي اليومية. |  |  |  |  |  |
| 3. | أستطيع أن أشارك أفراد عائلتي مخاوفي حول الغسيل الكلوي. |  |  |  |  |  |
| 4. | أفراد عائلتي على علم بحالتي الصحية. |  |  |  |  |  |
| 5. | أستطيع التحدث بصراحة إلى أفراد عائلتي عن تحدياتي مع الغسيل الكلوي. |  |  |  |  |  |
| 6. | يشجعني أفراد عائلتي على القيام بأنشطة صحية. |  |  |  |  |  |
| 7. | يقوم أفراد عائلتي بدعم قرارتي العلاجية |  |  |  |  |  |
| 8. | تتفهم عائلتي إمكانياتي المتعلقة بالغسيل الكلوي. |  |  |  |  |  |
| 9. | يقوم افراد عائلتي بتغيير نمط حياتهم لمساعدتي |  |  |  |  |  |
| 10. | يقوم أفراد عائلتي بدعم رفاهيتي بطريقة فعالة |  |  |  |  |  |

**الجزء الثاني: الدعم المجتمعي لمرضى غسيل الكلوي**

رحلة التعامل مع الغسيل الكلوي ليست مجرد رحلة فردية، بل هي أيضًا رحلة تتأثر بشكل كبير بفهم ودعم المجتمع؛ لذلك رؤيتك لدور المجتمع في تسهيل الرعاية، والتوعية، والتعاطف مع مرضى الغسيل الكلوي ذات قيمة كبيرة.

حدد مدى اتفاقك مع العبارات التالية:

| **العبارة** | | **غير موافق بشدة**  **(1)** | **غير موافق**  **(2)** | **محايد**  **(3)** | **موافق**  **(4)** | **موافق بشدة**  **(5)** |
| --- | --- | --- | --- | --- | --- | --- |
| 1. | أشعر أن مجتمعي يوفر لي الدعم العاطفي الذي أحتاجه للتعامل مع الغسيل الكلوي. |  |  |  |  |  |
| 2. | أعتقد أن الوعي المجتمعي حول الغسيل الكلوي قد حسَّن من جودة حياتي. |  |  |  |  |  |
| 3. | أتلقى مساعدة عملية من أعضاء المجتمع مما يجعل تحكمي في علاجي بالغسيل الكلوي أسهل |  |  |  |  |  |
| 4. | أشعر أنني أقل عزلة بسبب روابطي الاجتماعية مع مجتمعي. |  |  |  |  |  |
| 5. | يوفر مجتمعي موارد تعليمية تساعدني في فهم حالتي وعلاجي بشكل أفضل. |  |  |  |  |  |
| 6. | أجد أن الدعم من أقراني من مرضى الغسيل الكلوي في مجتمعي مفيد لصحتي النفسية. |  |  |  |  |  |
| 7. | أشعر ان مجتمعي يتعاطف معي بالتحديات التي أواجهها كمريض غسيل كلوي |  |  |  |  |  |
| 8. | أعتقد أن المبادرات المجتمعية قد سهلت عليّ الوصول إلى رعاية الغسيل الكلوي. |  |  |  |  |  |
| 9. | أشعر بالتشجيع من مشاركة المجتمع في رفع الوعي حول أمراض الكلى والغسيل الكلوي. |  |  |  |  |  |
| 10. | أجد أن الدعم المجتمعي قد أثر بشكل إيجابي على التزامي بنظام علاج الغسيل الكلوي. |  |  |  |  |  |

**الجزء الثالث: الدعم التنظيمي/المؤسسي للغسيل الكلوي**

تستطيع المؤسسات مثل المستشفيات والمشاركة التنظيمية أن تؤثر بشكل عميق على تجربة الأفراد الذين يتعاملون مع الغسيل الكلوي. لذلك نحن مهتمون بفهم تصورك للدعم الذي تقدمه المستشفى أو مركز الغسيل الكلوي وكذلك المنظمات الاجتماعية أو المجتمعية بشكل عام فيما يتعلق برعاية مرضى الغسيل الكلوي.

عبّر عن مدى اتفاقك مع العبارات التالية:

| **العبارة** | | **غير موافق بشدة**  **(1)** | **غير موافق**  **(2)** | **محايد**  **(3)** | **موافق**  **(4)** | **موافق بشدة**  **(5)** |
| --- | --- | --- | --- | --- | --- | --- |
| 1. | يوفر المستشفى/مركز الغسيل الكلوي لي تعليمًا شاملًا وموارد تعليمية شامله لفهم حالتي وعلاجي بشكل أفضل. |  |  |  |  |  |
| 2. | أشعر أن الموظفين في المستشفى/مركز الغسيل الكلوي يتعاطفون معي ويستجيبون لاحتياجاتي كأحد مرضى الغسيل الكلوي. |  |  |  |  |  |
| 3. | يوجد في المستشفى/مركز الغسيل الكلوي مجموعات أو برامج تساعدني في التغلب على التحديات العاطفية المرتبطة بالغسيل الكلوي. |  |  |  |  |  |
| 4. | أعتقد أن المستشفى/مركز الغسيل الكلوي يتعاون بفعالية مع المنظمات المجتمعية لرفع الوعي حول أمراض الكلى والغسيل الكلوي. |  |  |  |  |  |
| 5. | يبذل المستشفى/مركز الغسيل الكلوي جهودًا لإشراك عائلتي وأحبائي في خطة رعايتي وعلاجي. |  |  |  |  |  |
| 6. | أشعر بالرضا عن مستوى الرعاية والاهتمام الذي أتلقاه من المستشفى/مركز الغسيل الكلوي خلال جلسات الغسيل الكلوي. |  |  |  |  |  |
| 7. | أشعر أن المنظمات المجتمعية في منطقتي تعمل بنشاط على تعزيز الفهم والدعم لمرضى الغسيل الكلوي. |  |  |  |  |  |
| 8. | أعتقد أن المستشفى/مركز الغسيل الكلوي والمنظمات المجتمعية ملتزمون بتحسين جودة الحياة العامة لمرضى الغسيل الكلوي. |  |  |  |  |  |
| 9. | أشعر أن المستشفى/مركز الغسيل الكلوي والمنظمات المجتمعية قد أنشأوا بيئة داعمة تعزز الأمل والإيجابية لمرضى الغسيل الكلوي. |  |  |  |  |  |
| 10. | قدم لي المستشفى/مركز الغسيل الكلوي امكانية الوصول إلى الخدمات والاستشارات حول الصحة النفسية لمساعدتي في التعامل مع الأثر النفسي للغسيل الكلوي. |  |  |  |  |  |

**الجزء الرابع: السياسات الداعمة لمرضى الغسيل الكلوي**

تلعب السياسات دورًا أساسيًا في تشكيل الرعاية والدعم الذي يتلقاه مرضى الغسيل الكلوي. لذلك نريد تقييم تصورك لفعالية السياسات في معالجة قضايا الغسيل الكلوي في منطقتنا.

عبّر عن مدى اتفاقك مع العبارات التالية:

| **العبارة** | | **غير موافق بشدة**  **(1)** | **غير موافق**  **(2)** | **محايد**  **(3)** | **موافق**  **(4)** | **موافق بشدة**  **(5)** |
| --- | --- | --- | --- | --- | --- | --- |
| 1. | أعتقد أن السياسات الحالية تضمن أن أتلقى علاج الغسيل الكلوي في الوقت المناسب وبشكل كافٍ. |  |  |  |  |  |
| 2. | أشعر أن السياسات المعمول بها توفر وصولاً كافياً إلى الإمدادات الطبية الضرورية لجلسات الغسيل الكلوي الخاصة بي. |  |  |  |  |  |
| 3. | أنا واثق من أن السياسات تدعم توفر الكوادر الطبية المدربة لمساعدتي في الغسيل الكلوي. |  |  |  |  |  |
| 4. | أعتقد أن السياسات تتعامل بشكل فعال مع الحاجة إلى الدعم النفسي لمرضى الغسيل الكلوي. |  |  |  |  |  |
| 5. | أشعر أن السياسات تضمن التمتع بالوصول إلى الاستشارة والدعم الغذائي. |  |  |  |  |  |
| 6. | أعتقد أن السياسات تعزز الوعي وفهم الغسيل الكلوي داخل المجتمع. |  |  |  |  |  |
| 7. | أشعر أن السياسات المعمول بها تحمي حقوقي كأحد مرضى الغسيل الكلوي. |  |  |  |  |  |
| 8. | أعتقد أن السياسات سهلت تنسيق وتظافر الجهود بين المستشفيات والمنظمات المجتمعية لدعم مرضى الغسيل الكلوي. |  |  |  |  |  |
| 9. | أشعر أن السياسات تضمن أن أتلقى معاملة عادلة بغض النظر عن حالتي الاجتماعية والاقتصادية. |  |  |  |  |  |
| 10. | أعتقد أن السياسات تستجيب لاحتياجات وتحديات مرضى الغسيل الكلوي المتغيرة. |  |  |  |  |  |

**أشكركم على مشاركتكم**
