# Supplementary material for: Development and validation of the arabic version of the social-ecological model questionnaire for patients undergoing maintenance hemodialysis
Source: PLoS One. 2025 Oct 16;20(10):e0333740. doi: 10.1371/journal.pone.0333740 (PMC12530521; doi:10.1371/journal.pone.0333740)
Supplement: Attachment — Submitted filename: Reviewer_Comments.docx [file pone.0333740.s005.docx]

**Reviewer’s comments and Author’s responses**

**Comments to the Author**

1. If the authors have adequately addressed your comments raised in a previous round of review and you feel that this manuscript is now acceptable for publication, you may indicate that here to bypass the “Comments to the Author” section, enter your conflict of interest statement in the “Confidential to Editor” section, and submit your "Accept" recommendation.

Reviewer #1: (No Response)

Reviewer #3: All comments have been addressed

**Author’s response:**

Thank you

2. Is the manuscript technically sound, and do the data support the conclusions?
The manuscript must describe a technically sound piece of scientific research with data that supports the conclusions. Experiments must have been conducted rigorously, with appropriate controls, replication, and sample sizes. The conclusions must be drawn appropriately based on the data presented.

Reviewer #1: Partly

Reviewer #3: Yes

**Author’s response:**

Thank you, we have made the necessary change in the manuscript.

3. Has the statistical analysis been performed appropriately and rigorously?

Reviewer #1: No

Reviewer #3: Yes

**Author’s response:**

Thank you, we have made the necessary change in the manuscript.

4. Have the authors made all data underlying the findings in their manuscript fully available?
The [PLOS Data policy](http://www.plosone.org/static/policies.action#sharing) requires authors to make all data underlying the findings described in their manuscript fully available without restriction, with rare exception (please refer to the Data Availability Statement in the manuscript PDF file). The data should be provided as part of the manuscript or its supporting information, or deposited to a public repository. For example, in addition to summary statistics, the data points behind means, medians and variance measures should be available. If there are restrictions on publicly sharing data—e.g. participant privacy or use of data from a third party—those must be specified.

Reviewer #1: Yes

Reviewer #3: Yes

**Author’s response:**

Thank you.

5. Is the manuscript presented in an intelligible fashion and written in standard English?
PLOS ONE does not copyedit accepted manuscripts, so the language in submitted articles must be clear, correct, and unambiguous. Any typographical or grammatical errors should be corrected at revision, so please note any specific errors here.

Reviewer #1: No

Reviewer #3: Yes

**Author’s response:**

Thank you.

**Reviewer #1:**

**Introduction**
The background offers a general justification for the research, highlighting the need for psychosocial support during hemodialysis and the necessity of context-specific measurement tools among Arabic-speaking populations. It utilises the Social-Ecological Model (SEM) as the theoretical framework to inform the development of the questionnaire.

• The introduction can also be enhanced by the clear stipulation of research questions and goals, ideally as a last paragraph to ground the study.

• The reasoning for the application of SEM in particular—apart from its multi-level aspect—must be justified with excerpts of previous empirical use of SEM in the treatment of chronic disease or dialysis. Include a concluding paragraph in the introduction that clearly outlines the study’s aim, objective, and research importance to maintain greater coherence and guide the reader.

**Author’s response:**

In the last two paragraphs, we have justified the application of the social ecological model (SEM). We believe that the SEM provides a comprehensive framework that enables a multidimensional evaluation of support from various angles. By integrating various layers of influence, including individual, interpersonal, community, and societal factors, the SEM helps capture a broad and nuanced understanding of the context. This holistic approach not only considers intrapersonal dynamics but also the broader environmental and societal conditions that may impact outcomes. Such an inclusive perspective enables a more thorough assessment and better-informed decision-making.

**Reviewer #1:**

Literature Review
The literature review tells us about the SEM and the hemodialysis burden but fails to link these observations to each other or to place properly the need for a new instrument or adaptation.
• The relationship between SEM areas and burden of dialysis is under-explored. For example, what effect do organizational-level support have on outcomes for MHD? Incorporate empirical studies that utilize SEM to investigate compliance or chronic illness management (e.g., HIV, diabetes, ESRD).
• Lacking regional specificity—no distinct references to existing health literacy or psychosocial research on Arabic-speaking hemodialysis patients. Add Arab-world or Middle Eastern-specific research of support systems or barriers in treatment.
• Neglects to mention comparable validated instruments (e.g., MOS-SSS, MSPSS) and how the SEM-based instrument varies or is superior. Provide a concise critique of current measures of assessment and reasoning of support, which account for why a SEM-based measure is theoretically superior or more fitting.

**Author’s response**

The second and third paragraphs addressed the reviewer’s comments regarding the literature review. However, further elaboration has been made to provide a comparative justification for other scales used in measuring social support.

**Reviewer #1:**

**Methodology**
The research employs the typical psychometric validation process, which consists of content validity, face validity, exploratory factor analysis (EFA), reliability testing, and convergent/discriminant validity testing.
• The cultural translation and adaptation are not provided with critical details. For example, how variations in forward–backward translation were managed is not specified. Define translator qualifications and whether pilot test or panel review was done.

• ICC face validity testing is not adequately described; there is no ICC model (e.g., ICC(2,1)) and no confidence intervals. Identify the ICC model used, include confidence intervals, and explain threshold interpretations.

• Sampling strategy is not justified—no reason why the sample size was chosen or whether power was taken into account. Clarify the sample size on the basis of acceptable psychometric practice (e.g., participants per item, power analysis).

Statistical analyses include descriptive statistics, EFA, and reliability/validity statistics. These are suitable for stage-one scale validation but are short of some assumption testing and transparency.

• EFA's assumptions (e.g., multivariate normality, linearity) are not tested or stated. Test and report multivariate normality if Maximum Likelihood extraction is employed.

• Use parallel analysis or scree plot inspection to make an argument for the number of factors retained. • Report correlation and factor loading matrices to allow transparency. • Retention of factors is eigenvalue and explained variance based only. Parallel analysis and other stricter criteria are not employed.

• Fornell–Larcker and HTMT thresholds are noted but not interpreted.
• Place all validity/reliability measures into a single table for ease of interpretation.

• Tabular summaries of factor loadings and communalities are not presented.
• Graphical aids (e.g., scree plot, histograms of item distributions) are not included to aid interpretation.

• Measurement Invariance: No subgroup analysis (e.g., gender, age) even with an imbalanced sample.

• Minimal explanation of why certain items (e.g., Pol_10, Org_5) underperformed and conceptual implications.

**Author’s response**

All comments were thoroughly reviewed, reanalyzed, and revised to ensure clarity and ease of understanding by providing appropriate values. Although a multigroup analysis was not conducted, we focused on analyzing the gender differences in Cronbach's alpha. This analysis revealed no statistically significant results, indicating uniformity and consistency across genders. The rewritten texts are marked with blue fonts.

**Reviewer #1:**

Discussion and Conclusion
The discussion is in terms of the study purposes and SEM model and recognizes novelty and usability of the validated tool but is devoid of theory and limitation consideration.
• The limited predictive ability of the "Policy Support" factor is not addressed in detail. Explain why questions related to policy may have underperformed (e.g., cultural confusion, insufficient policy knowledge).
• Low degree of engagement with cultural or systems-level implications of results. Consider cultural conceptions of support of Arab hemodialysis patients.

• Limitations section can be amended to detail the sample's features (e.g., gender bias) and cross-validation requirements. Suggest a cross-group validation (e.g., education, gender) and confirmatory factor analysis (CFA) in subsequent research.
• Confirmatory Analysis: Because it is exploratory research, CFA is understandably missing. Nevertheless, this should be discussed and suggested to be performed in the future.

**Author’s response**

1. Policy Support Factor—Theoretical and Cultural Considerations
The discussion has been revised to provide a more detailed theoretical explanation for the limited predictive ability of the "Policy Support" domain. The manuscript now describes how patients in Arabic-speaking contexts, especially in Gaza, often perceive policy-related influences as abstract due to systemic factors such as fragmented health governance, limited policy literacy, and sociopolitical instability. As a result, respondents may have lower awareness of or less engagement with policy-level determinants, reducing the relevance and clarity of these questionnaire items. This may account for the weaker psychometric performance of the Policy Support factor. These points and relevant literature are now reflected in the revised discussion and limitations sections.

2. Cultural and Systems-Level Implications
Additional content has been incorporated to discuss how family and community support are deeply central within Arab collectivist cultures, particularly for patients with chronic illness such as MHD. The discussion elaborates on the cultural expectations around support—highlighting the tendency to rely on interpersonal, community, and organizational resources over impersonal policy-level interventions. This extends the cultural interpretation of results and situates findings in the broader context of Arab and Middle Eastern health systems.

3. Limitations—Sample Features and Cross-Validation
The limitations section has been extended to address the sample’s composition, including gender and educational biases. The need for cross-group validation—by gender, education, and other sociodemographic factors—has been clearly recommended for future research. These recommendations aim to enhance the generalizability of the findings.

4. Confirmatory Factor Analysis (CFA) and Future Research
The manuscript clarifies that given the exploratory nature of the current study, confirmatory factor analysis (CFA) was not performed. However, the importance of CFA is acknowledged, and explicit suggestions for future studies to conduct CFA, as well as to undertake longitudinal and cross-national validation (including other Arab countries), have been included.

We are grateful for these comments, which have helped to strengthen the theoretical depth, cultural relevance, and methodological transparency of our discussion and recommendations. All suggested changes are now reflected in the revised manuscript.

Reviewer #3:

Thank you for the opportunity to review this manuscript. I believe all comments have been addressed.

**Author’s response**

Thank you very much.
